# Anti-inflammatory Gold-Induced Autologous Cytokines treatment triggers heart failure after myocardial infarction

**DOI:** 10.15190/d.2017.10

**Published:** 2017-12-31

**Authors:** Franziska Cordes, Adelina Curaj, Sakine Simsekyilmaz, Ulrich Schneider, Elisa A. Liehn

**Affiliations:** ^1^Institute for Molecular Cardiovascular Research (IMCAR), RWTH Aachen University, Germany; ^2^Victor Babes National Institute of Pathology, Bucharest, Romania; ^3^Biochemistry Institute, Justus-Liebig-University, Giessen, Germany; ^4^Arthro Nova Clinic, Ringsee, Germany; ^5^Human Genetics Laboratory, University of Medicine and Pharmacy, Craiova, Romania

**Keywords:** GOLDIC, cytokines, myocardial infarction, inflammation

## Abstract

Background: Gold-induced autologous cytokine (GOLDIC) treatment is usually used in the therapy of the inflammatory musculoskeletal disorders (e.g. osteoarthritis in humans) and is able to modulate the inflammatory reaction. Moreover, governed by chemokines and cytokines, the complex inflammatory response after an acute myocardial infarction (MI), the main cause of death worldwide, plays an important role in the preservation of heart function. Therefore, we hypothesized that GOLDIC could also have an important role in ventricular remodeling after MI. Methods: Myocardial infarction was induced in mice and GOLDIC-enriched serum was directly injected directly in the infarcted tissue. Four weeks later, the function of the heart, as well as the infarction size and the scar composition were analyzed. Statistical analysis was performed with Prism 6.1 software (GraphPad), using 1-way ANOVA, followed by Newman-Keuls post-hoc-test, as indicated. Data are represented as mean ± SEM. Results: Four weeks after MI, GOLDIC-treated mice show significantly decreased heart function and higher infarction size compared to the control group. Immunohistochemistry reveals a significantly increased number of myofibroblasts, correlating with higher collagen content in the infarcted area. Despite impaired heart function, angiogenesis in the GOLDIC-treated group is improved compared with the control, due to the increased vascular endothelial growth factor (VEGF) in the GOLDIC serum. Conclusions: In conclusion, GOLDIC treatment impairs the ventricular remodeling, worsening the heart function. Therefore, these systemic effects should be taken into account when new therapies are designed for the musculoskeletal disorders.

## INTRODUCTION

As one of the most frequent causes of morbidity and mortality in western countries, myocardial infarction (MI) triggers a complex inflammatory response, governed by different chemokines and cytokines^1^. These various factors recruit immune cells, which are responsible for the initiation, the control and the modulation of repair processes, and the remodeling of the injured heart tissue after an infarction^2^. The mechanism behind this progress defines the scar formation and, consequently, the cardiac function after MI.

Gold has been used for centuries in ancient Egypt and China for the treatment of different mental, spiritual, and physical diseases. Recently, its beneficial effects in patients suffering from rheumatoid arthritis were proved^3^. Direct administration of gold particles suppressed NF-kappa B pathway activation, reduced production of proinflammatory cytokines, such as TNF-alpha, interleukin-1 and interleukin-6, and enhanced secretion/production of anti-inflammatory factors, such as interleukin-4^4,5^. However, many patients exhibit adverse immune-mediated side effects, such as allergy, renal, skin or oral mucosal complications, necessitating an immediate interruption of the therapy.

Small gold particles incubated with blood lead to an enrichment in different cytokines in the serum. The main effect of this Gold-induced autologous cytokines (GOLDIC) procedure is the significant increase of gelsolin^6^, a protein responsible for the regulation of actin filaments in particular, and cell structure and metabolism in general^7^. Thus, it is supposed to decrease the side effects^8^. GOLDIC are used intra-articular in the treatment of musculoskeletal disorders, such as osteoarthritis, modulating successfully the inflammatory response^8^.

Therefore, we hypothesized that GOLDIC might be able to influence the inflammatory reaction and to significantly contribute to the ventricular remodeling after MI.

## MATERIALS AND METHODS

### Production of Serum

The mouse blood was collected in a tube and pretreated with gold particles according to the manufacturer´s protocol (Arthrogen GmbH, Germany)^6^. Briefly, the blood was incubated at 37°C for 24 hours. After centrifugation at 5000rpm for 10 minutes, the serum containing gold-induced autologous cytokines was extracted. Untreated serum served as control.

### Myocardial Infarction Induction and Treatment

Eight to ten weeks old male C57Bl/6N mice were intubated under general anesthesia (100 mg/kg bodyweight ketamine, 10 mg/kg bodyweight xylazine, i.p.) and analgesia (0,1 mg/kg bodyweight buprenorphine, s.c.). They were ventilated using a rodent respirator with positive-pressure and oxygen. Isoflurane was administered at a dose of 0,25-2ml/min during the procedure to assure proper anaesthetization. A left side thoracotomy was performed and MI was induced by permanent ligation of the proximal left anterior descending artery^9^. After MI induction, visible by a to-grey colour-change of the affected heart tissue, 30µl GOLDIC-enriched or control serum was injected into the border zone using a 33-gauge needle. The ribs, muscle and skin incisions were closed with separate sutures. The analgesia was continued for five days after MI induction by daily injection of 0,1mg/kg bodyweight buprenorphine subcutaneously. All animal experiments and study protocols were approved by local authorities (Landesamt für Natur, Umwelt- und Verbraucherschutz Nordrhein-Westfalen, reference number: 84-02.04.2013.A185), complying with European and German animal protection laws.

### Echocardiography

Before and four weeks after MI induction, left ventricular heart function was assessed by echocardiography performed on a small-animal ultrasound imager (Vevo 770, FUJIFILM Visualsonics, Toronto, Canada). Measurements were recorded in B-Mode (2D-realtime) and M-Mode. During the procedure, the mice were anaesthetized by inhalation narcosis, with 2ml/min isoflurane. The ejection fraction (EF), fractional shortening (FS), cardiac output (CO), heart rate (HR) and left ventricular mass were recorded and analyzed using the VisualSonics Software.

### Histology and immunohistochemistry

Four weeks after MI induction, infarction size was evaluated. Mice were anaesthetized (100 mg/kg bodyweight ketamine, 10 mg/kg bodyweight xylazine, i.p.). Hearts were excised, fixed in formalin and embedded in paraffin. Serial sections (10-12 sections per heart, 400 µm apart, up to the mitral valve) were stained with Gomori’s 1-step trichrome stain. The infarcted area was determined for all sections using Diskus software (Hilgers, Königswinter, Germany) and expressed as percentage of total left ventricular volume. Blue-stained collagen content was analyzed with Cell P Software (Olympus, Hamburg, Germany) and expressed as a percentage of the infarcted area. Additional sections (3 sections per heart, 400 µm apart) were stained to analyze within the infarcted area the content of myofibroblasts (smooth muscle actin, DAKO, Germany) and CD31-positive capillaries (CD31, Santa Cruz, Santa Cruz, CA). Positive-stained cells or vessels were counted in six different fields per section and expressed as cells or vessels per mm^2^.

### ELISA

Enzyme Linked Immunosorbent Assay (ELISA) was performed from serum samples using the DuoSet ELISA Development System kit for mouse Keratinocyte-derived chemokine (KC) (R&D Systems), vascular endothelial growth factor (VEGF), stromal derived factor (SDF)-1 and monocytes chemotactic protein (MCP)-1.

### Statistical analysis

Data are represented as mean ± SEM. Statistical analysis was performed with Prism 6.1 software (GraphPad). Means of two groups were compared with unpaired Student-t test, while for more than two groups, 1-way ANOVA followed by Newman-Keuls post-hoc-test were used, after passing D'Agostino and Pearson omnibus normality test, as indicated. P-values of <0.05 were considered significant.

## RESULTS

**Figure 1:**
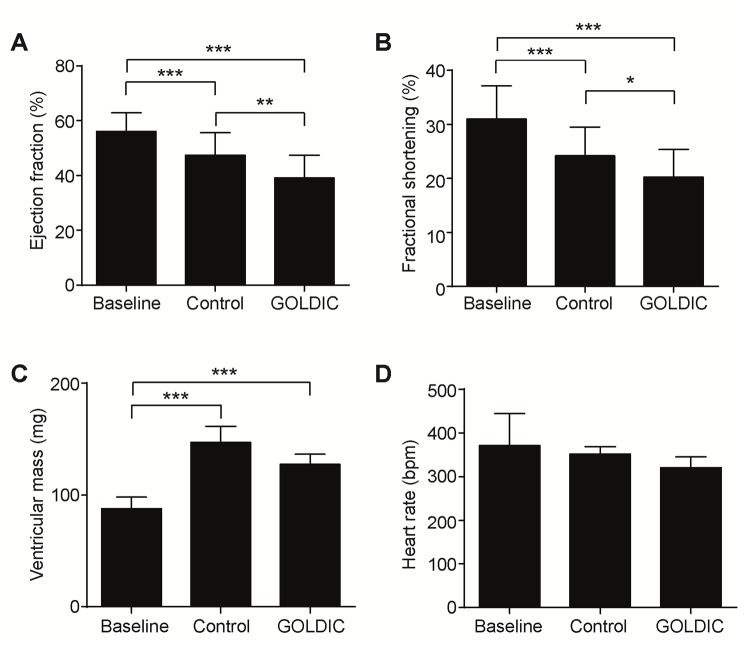
Echocardiographic measurements (A) Ejection fraction measured before (baseline) as well as four weeks after MI showed significant reduction in GOLDIC-treated mice compared to control group. (B) Fractional shortening confirms the reduced pump function of the GOLDIC-treated group. (C) Ventricular mass was reduced after MI, but did not differ between the experimental groups. (D) Heart rate was constant in all mice during the experiment.

**Figure 2:**
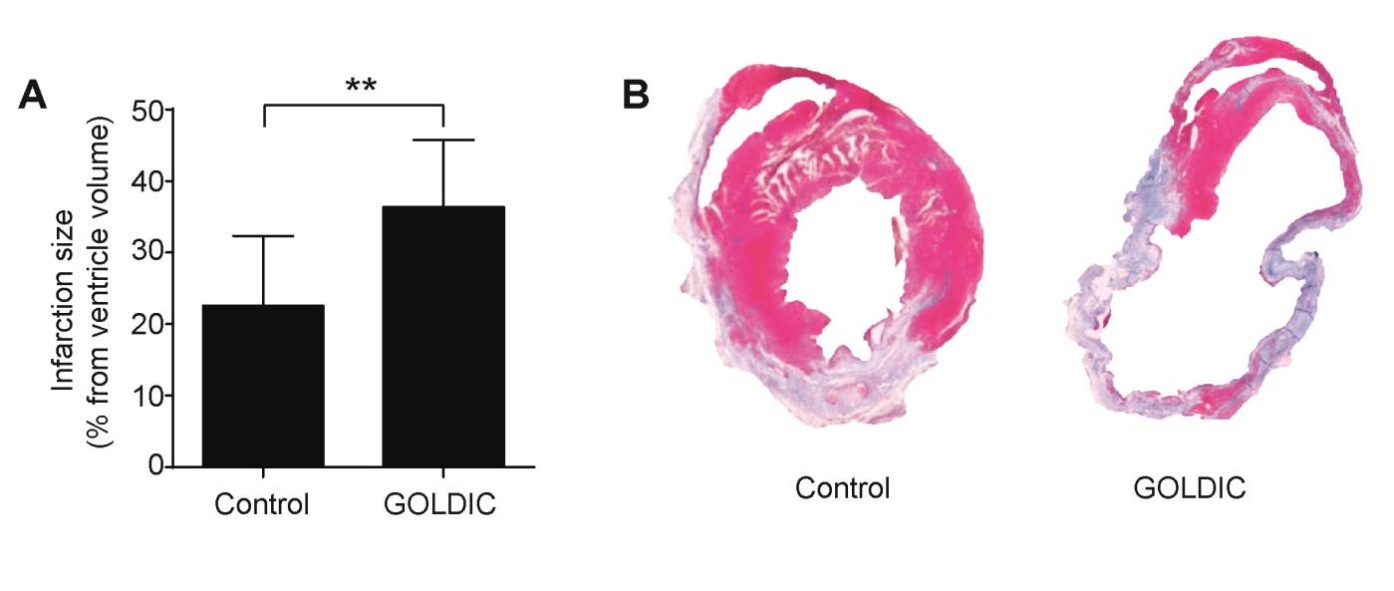
Histological assessment of infarction size (A) Infarction size was significantly increased in the GOLDIC-treated mice compared to the control group. (B) Representative images of Gomori’ staining from control and GOLDIC-treated mice (red - viable myocardium, blue - infarction scar).

Before and 4 weeks after MI induction and GOLDIC injection, the cardiac function was measured by echocardiography. The ejection fraction (**Figure 1** [A]) was significantly decreased in the GOLDIC-treated group (39.2 ± 1.7%, n=8) compared to the control group (47.5 ± 1.6%, n=7, p<0.05), as well as the fractional shortening (**Figure 1** [B], 20.2 ± 1.2% vs. control 24.2+-1.1%, p<0.05) in the GOLDIC-treated group in comparison to the control group. The V ventricular mass (**Figure 1** [C]), heart rate (**Figure 1** [D]) and cardiac output (11.8 ± 1.6 ml/min vs. control 15.5 ± 0.9 ml/min) did not differ between the groups after MI induction. All these data pointed out an unexpected reduction of left ventricular heart function after GOLDIC-treatment. The echocardiographic results were confirmed by histological analysis of the left ventricular infarction size, which was significantly higher in GOLDIC-treated mice than in the control group (36.4 ± 2.9%, n=8, vs. control 22.6 ± 2.8%, n=7, p<0.05, **Figure 2**).

**Figure 3:**
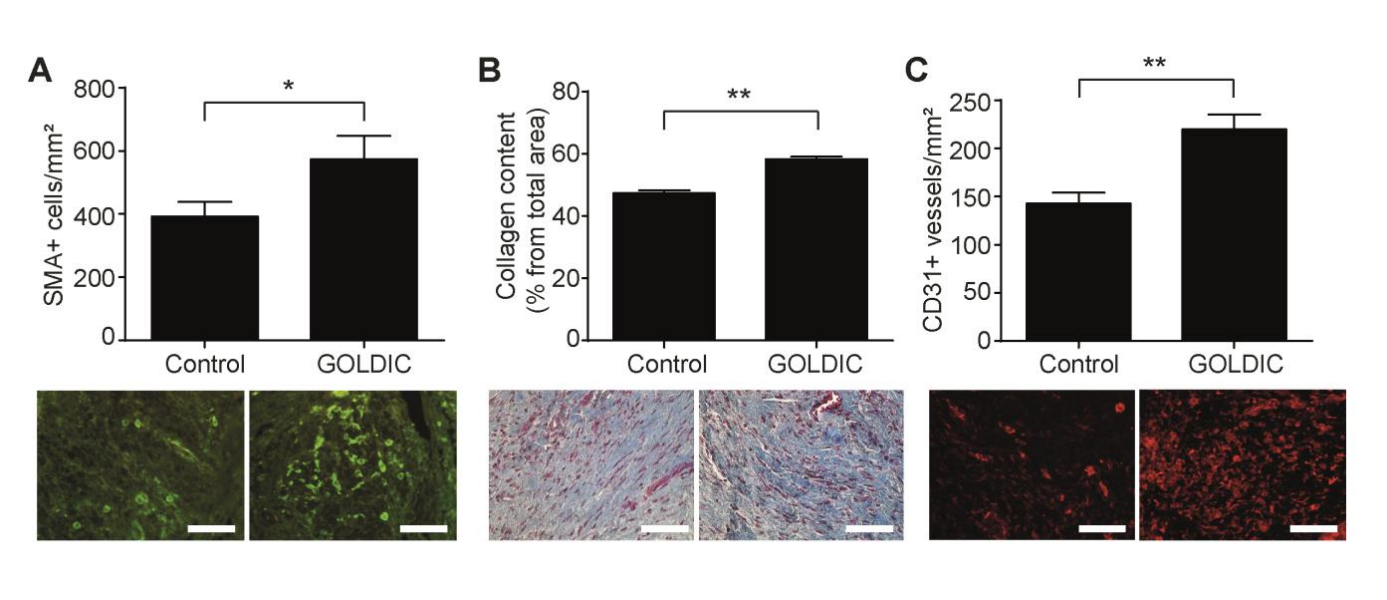
Immunohistological analysis of the infarcted area (A) Number of myofibroblasts increased in the GOLDIC-treated group compared to the control group, as shown by the smooth muscle actin positive staining (green, scale bar 50 µm). (B) This was associated with increased collagen content (blue, scale bar 50 µm). (C) Angiogenesis determined by counting CD31 positive newly-formed vessels is increased in GOLDIC-treated mice compared to the control group (red, scale bar 50 µm).

Immunofluorescent staining of smooth muscle actin positive cells showed that the number of myofibroblasts was significantly increased in the GOLDIC-treated group, associated with a higher content of collagen in the scar (**Figure 3** [A]). Surprisingly, we found an increase in angiogenesis (CD31 positive newly-formed vessels) in the GOLDIC-treated group (6.0 ± 0.5 vessels/field, p<0.05) compared to the control mice (9.2 ± 0.7 vessels/field, p<0.05, **Figure 3** [B]). This was associated with high levels of Vascular Endothelial Growth Factor (VEGF, 555±50 pg/ml) and Stromal-cell Derived Factor (SDF-1, 93±2 pg/ml), in the GOLDIC-serum, measured by ELISA.

## CONCLUSION

In this study, mice were injected with GOLDIC-serum into their injured myocardium after MI induction. Four weeks after the procedure, we found an impaired ventricular remodeling and worsening of the heart function after GOLDIC-treatment, despite increased angiogenesis.

These results sustain the thesis that anti-inflammatory therapies are not always beneficial for the healing of the heart after MI. It was always believed that reducing the initial inflammation, mostly induced by neutrophils, should improve the ventricular scar remodeling and preserve the heart function^1^. However, more and more new data demonstrate that impairing the neutrophil recruitment might have double-edge effects, since it interferes with the healing response, polarizing macrophages towards a reparative phenotype^10-12^. Neutrophil depletion in mice induces an increased myofibroblasts proliferation and collagen content, associated with impaired heart function^11^, which we also observed in the present study. Moreover, in clinical studies the use of anti-inflammatory therapies caused impaired wound healing and increased cardiac rupture, despite a decrease in tissue damage^13,14^- a situation which forces physicians to be more careful when anti-inflammatory strategies are designed for patients suffering an acute myocardial infarction. In the present study, we demonstrate that the direct transplantation of GOLDIC-enriched serum into the heart exacerbates the worsening effects. However, we pointed out possible and important side effects of the anti-inflammatory therapy, which should be critically scrutinized during treatment to avoid severe complications, especially in patients with cardiac disease.

Interestingly, we found an increased angiogenesis in the infarcted area after GOLDIC treatment. In general, it was believed that increased angiogenesis would automatically preserve or improve heart function after MI. Therefore, many therapies were developed to increase the vessel formation in the infarcted area^15-18^. However, some studies demonstrate that there is no direct correlation between the neo-angiogenesis and the improved heart function^12,19^. Thus, we conclude for this study, that the pro-angiogenic factors induced by the GOLDIC-procedure in serum have no beneficial effects on the healing after MI. Furthermore, due to technical reasons, main components of GOLDIC-treated serum identified in human (Gelsolin, G-CSF, S-CSF) could not be determined in mouse, therefore, the applicability of the results is probably critical to the human situation.

In conclusion, since GOLDIC seems to be beneficial in the treatment of many inflammatory diseases, the negative effects on the heart function should be taken into account, when systemic therapies with GOLDIC-serum are performed.
